# Straightforward model construction and analysis of multicomponent biomolecular systems in equilibrium†

**DOI:** 10.1039/d2cb00211f

**Published:** 2023-01-18

**Authors:** Nick H. J. Geertjens, Pim J. de Vink, Tim Wezeman, Albert J. Markvoort, Luc Brunsveld

**Affiliations:** a Laboratory of Chemical Biology, Department of Biomedical Engineering and Institute for Complex Molecular Systems, Eindhoven University of Technology, Den Dolech 2 Eindhoven 5612 AZ The Netherlands l.brunsveld@tue.nl; b Computational Biology Group, Department of Biomedical Engineering and Institute for Complex Molecular Systems, Eindhoven University of Technology, P.O. Box 513 Eindhoven 5600 MB The Netherlands A.J.Markvoort@tue.nl

## Abstract

Mathematical modelling of molecular systems can be extremely helpful in elucidating complex phenomena in (bio)chemistry. However, equilibrium conditions in systems consisting of more than two components, such as for molecular glues bound to two proteins, can typically not be analytically determined without assumptions and (semi-)numerical models are not trivial to derive by the non-expert. Here we present a framework for equilibrium models, geared towards molecular glues and other contemporary multicomponent chemical biology challenges. The framework utilizes a general derivation method capable of generating custom mass-balance models for equilibrium conditions of complex molecular systems, based on the simple, reversible biomolecular reactions describing these systems. Several chemical biology concepts are revisited *via* the framework to demonstrate the simplicity, generality and validity of the approach. The ease of use of the framework and the ability to both analyze systems and gain additional insights in the underlying parameters driving equilibria formation strongly aids the analysis and understanding of biomolecular systems. New directions for research and analysis are brought forward based on the model formation and system and parameter analysis. This conceptual framework severely reduces the time and expertise requirements which currently impede the broad integration of such valuable equilibrium models into molecular glue development and chemical biology research.

## Abstract 2

A framework for equilibrium models, such as molecular glues and other contemporary biomolecular multicomponent systems, is presented that automatically generates custom mass-balance models based on the reversible reactions of each system.

## Graphical abstract

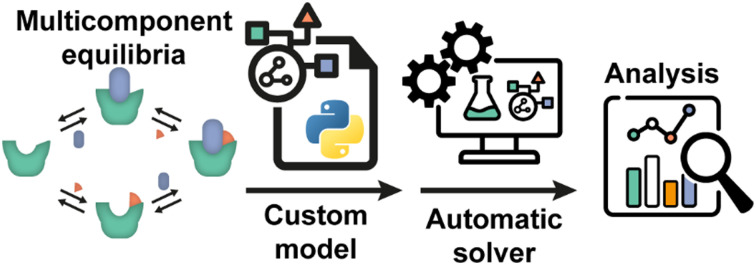

## Introduction

Interactions between molecules and their resulting assemblies are central features of biological and chemical systems and understanding its fundamentals is crucial in the chemical sciences.^1,2^ In chemical biology, the understanding of the formation of multicomponent biomolecular assemblies, such as those formed *via* PROTACs,^3^ molecular glues,^4^ scaffold proteins,^5^ antibodies,^6^ and alike supports the development of next generation therapeutics. In addition, complex biomolecular phenomena involving nonlinearity such as competition, self-sorting,^7^ crosstalk,^8^ scaffolding,^5^ templating,^9^ cooperativity,^10^ multivalency,^11^ and ultrasensitivity^12^ often require the aid of mathematical models for detailed analysis and understanding of the crucial molecular interactions involved. In turn, the use of (thermodynamic) computational models has gained popularity to deduce binding mechanisms involved, design experiments, analyse data and determine system constants.^13^

The solution to the equilibrium distribution of a two-component biomolecular system, with one-to-one binding, goes as far back as the Langmuir adsorption model, however more complicated systems consisting of three or more components can only be solved analytically in certain specific cases^14,15^ or after additional assumptions.^16^ The ternary body problem is mathematically unsolvable without approximation or assumption of the free concentration of one of the components.^17^ It has therefore gotten more common to develop custom-made mathematical models for specific types of biomolecular systems based on a combination of analytical and numerical solutions.^17–20^ Such equilibrium models have also been used for analysis of combinatorial libraries,^21^ macromolecular reactions within living cells,^22^ supramolecular copolymerization,^23^ and the design of synthetic signalling pathways.^24^ However, the creation of a new model for each unique system takes expertise and a non-trivial amount of time. The widespread integration of these models into chemical biology is thus impeded, even though a trove of information can be extracted from them. Increasing the accessibility of numerical equilibrium models will greatly aid the study of complex biomolecular systems.

Here we present a framework for multi-equilibrium models as a general approach to generating a custom model for any biomolecular system based on the simple, reversible reactions that constitute that system. The framework automatically determines the relations between the species concentrations at equilibrium and combines these with mass balance equations to analytically establish a system of coupled expressions for the equilibrium concentrations that are subsequently solved numerically, without the need to rely on assumptions, simplifications, and/or the need of computationally expensive calculations of reaction kinetics. In addition, the framework facilitates standardized methods for system and parameter analysis. This general approach severely reduces both the expertise and time required to construct new computational models. The framework was designed to especially cater to the experimental (laboratory) chemical biologist, who is possibly less accustomed to programming and modelling techniques. The manuscript starts with a description of the general derivation method. Next, this is used to revisit three chemical biology systems from recent literature^25,26^ to illustrate the simplicity and generality of the framework while providing models of the same quality as models created specifically for the cases in question, but also providing novel scientific insights in the underlying biomolecular interaction network. The complexity of the three systems is step-wise increased from a simple one-to-one protein–protein interaction *via* molecular glues for stabilization of protein–protein interactions^26^ to highly complex, multicomponent homogeneous immunoassays.^25^ The obtained parameters are compared to the original findings and the chemical biology systems at hand are analysed in more depth using methods from the framework, providing valuable insights and new directions for research. Installation instructions and complete protocols for the framework, as well as detailed tutorial descriptions to follow along with each of the described chemical biology cases are available in the ESI.†

## Methods

The framework is constructed entirely in Python and is freely available from DOI https://doi.org/10.5281/zenodo.5303678. Installation instructions can be found in ESI† Appendix, Section 1. The solver section uses an object-oriented approach and supports modification of parts for specific use cases while the analysis section uses separate functions to facilitate addition of custom analysis functions with automatic integration into the rest of the framework. Users familiar with Python can easily add specific analyses appropriate for their research as needed (ESI† Appendix, Section 10). The SymPy^27^ library is used to convert, using symbolic mathematics, the specified equilibrium reactions into a set of coupled equations for the equilibrium concentrations, whereafter these equations are numerically solved using the NumPy^28^ and SciPy^29^ libraries. When fitting to data, parameters are determined using the iterative scipy.optimize.least_squares solver with the ‘Trust Region Reflective’ algorithm.^29^ In addition, by default a log transform^30,31^ is applied to all parameters to support large order-of-magnitude differences between parameters and to increase the solving speed. The error function is defined as the mean squared error between the experimental values (averaging technical repeats) and the predicted values from the data-function for each titrate concentration. Multiple methods are implemented in order to solve the equilibrium equations (model.system_equations) for a specific titrate concentration. The solver will first attempt to use the faster but less robust scipy.optimize.root method before escalating to a least-squares approach in case no physical equilibrium can be determined. Based on the solution, all species concentrations are updated to the new equilibrium in the state object (model.update_state) and the data-function (model.data_function) converts species concentrations to an experimental data value prediction which is used in the error function. Stress tests have been performed to test the limits of the framework (ESI† Appendix, Section 11). Systems of up to a hundred unique species were tested and produced solutions in a reasonable time span.

## Results and discussion

### General approach

**Fig. 1:**
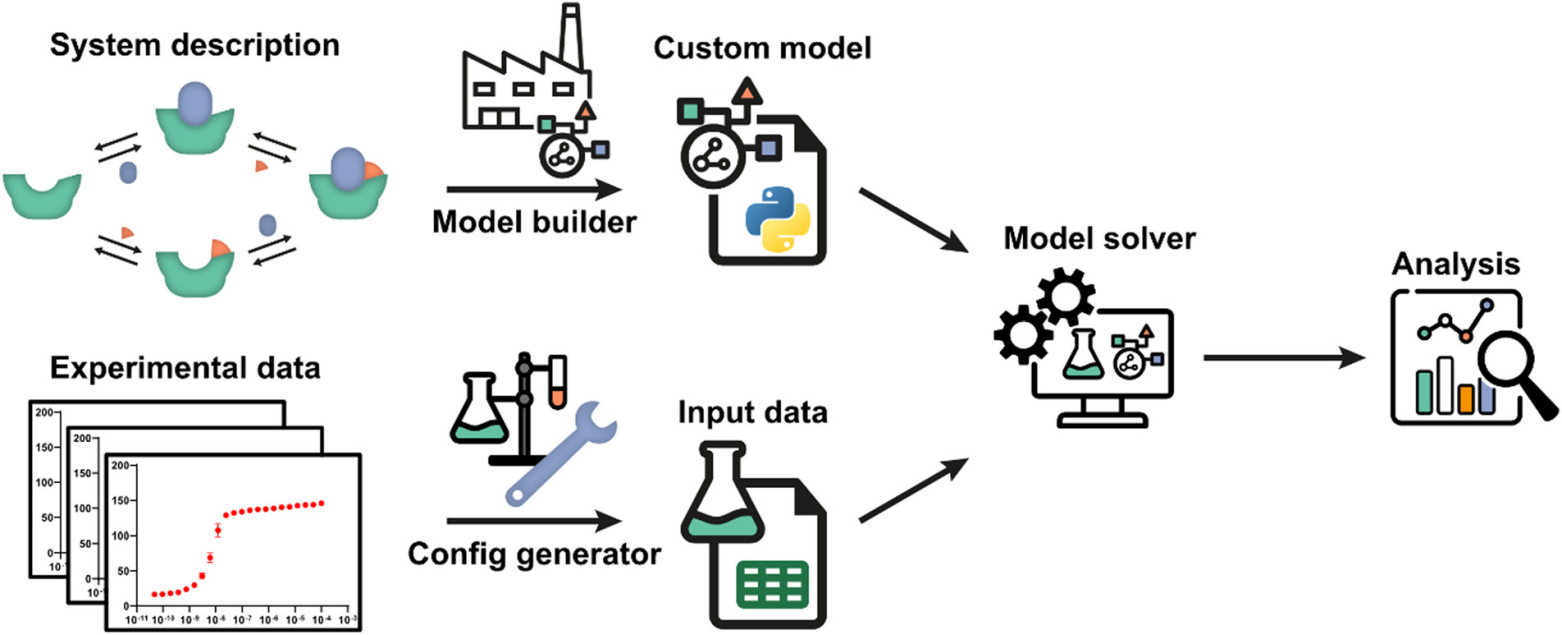
Schematic overview and workflow of the framework. The system description, a set of reversible reactions that describe how all species in the biomolecular system interact, is translated to a custom model by the model builder. After defining the experimental conditions using the config generator, experimental data can be used by the model solver in order to determine point estimates for any unknown parameters in the custom model. Subsequently, the custom model can be analysed by the framework. A number of diverse, extendible, and customizable methods were developed.

The framework utilizes an automatic model building process, based on a general symbolic derivation method, in order to streamline the creation of new multi-equilibrium models and provides options to analyse these models and to fit them to experimental data. A schematic overview of all steps and the workflow are depicted in Fig. 1. Starting point is a set of reversible reactions, as reversible reactions are a familiar and natural way to describe biomolecular interactions in a system. Combined, these reversible reactions form the system description, defining the system and all complexes that are formed therein. In a system at equilibrium, each reversible reaction is also in equilibrium and the ratio of species on both sides of each reaction is determined by the equilibrium constant.^32^ Therefore, the entire system can be described in terms of the equilibrium constants and the free concentrations of species that cannot disassociate further, designated the components of the system. The model builder determines an analytical expression for the equilibrium concentration of each complex in the system based on the equilibrium constant and concentrations of the complexing species. These expressions are automatically reworked until they contain solely the component concentrations and equilibrium constants.

For each single component within the system a mass balance equation is set up, stating that the overall concentration of that component should be equal to the equilibrium concentration of that component in uncomplexed form plus the sum of the equilibrium concentrations of all complexes multiplied by the number of times that component is present therein. Substituting the equilibrium expressions of the complexes determined earlier in these mass-balance equations results in a system of *n* equations with the *n* free component concentrations as the only unknowns. For given overall concentrations and equilibrium constants, these equilibrium equations can then be solved numerically by the model solver. All steps are performed autonomously based on the entered reversible reactions. A comprehensive protocol for each step in the framework (Fig. 1) is available in ESI† Appendix, Section 1.

Once a custom model has been generated, not only can it be solved for fixed concentrations and equilibrium constants, but also various analyses can be performed in the framework to gain insight into the biomolecular system at hand. These analyses include plotting the concentrations of all species in the system as a function of component concentrations for given equilibrium constants and parameter sensitivity analysis. Similarly, the framework allows predicting of equilibria upon tuning of equilibrium constants and concentrations, thus aiding in assay development.

The framework can also determine unknown parameters by fitting experimental data using the model solver. For this, a data function has been added that relates concentrations of one or more species to an experimental observable. The data function is specified in the model builder and is included in the custom model. The experimental data values must be a direct function of one or more species concentrations.^13,32^ Experiments where this is the case often include titrations and we will use the related terminology in the given examples. Experimental conditions in which the data was obtained can be defined with help of the config generator. Detailed instructions and the expected data format are described in the framework protocol (ESI† Appendix, Section 1). Using initial guesses for the unknown parameters, the model solver can then numerically calculate the equilibrium concentrations of all species based on the specified total concentrations for each data point. Subsequently, the values of the unknown parameters are iteratively adjusted to best fit the entire experimental dataset using a numerical least-squares optimization. The framework also offers various analyses for the critical assessment of the correctness of the chosen system description, *i.e.*, the specified equilibrium reaction(s), and of the quality of the determined parameter estimates for that model, including model prediction plots, mean-squared-error landscapes and confidence intervals determined using the bootstrap method. A complete overview of all analyses is available in ESI† Appendix, Section 2 and the most important ones are illustrated in the following case studies.

### Protein–protein interaction

Protein–protein interactions (PPI) are one of the main biological regulatory mechanisms and their study lies at the heart of chemical biology.^33^ 14-3-3 proteins are a family of highly preserved scaffold proteins that interact with hundreds of distinct protein partners.^34^ These hub proteins are involved in processes such as cell signalling, protein trafficking, cell cycle progression and apoptosis.^35,36^ Because of this, 14-3-3 proteins are closely involved with a number of human diseases and have proven to be interesting drug targets. The PPI between 14-3-3 and TASK3 regulates the activity of the TASK3 potassium ion channel^37^ and serves as an easy entree to introduce and demonstrate the framework approach, as such one-to-one interactions can also be solved analytically.^38^ Fluorescent anisotropy data^39^ provides the input to determine the dissociation constant (*K*_D_) for the interaction between 14-3-3 and TASK3 (Dataset S1, ESI†).

**Fig. 2:**
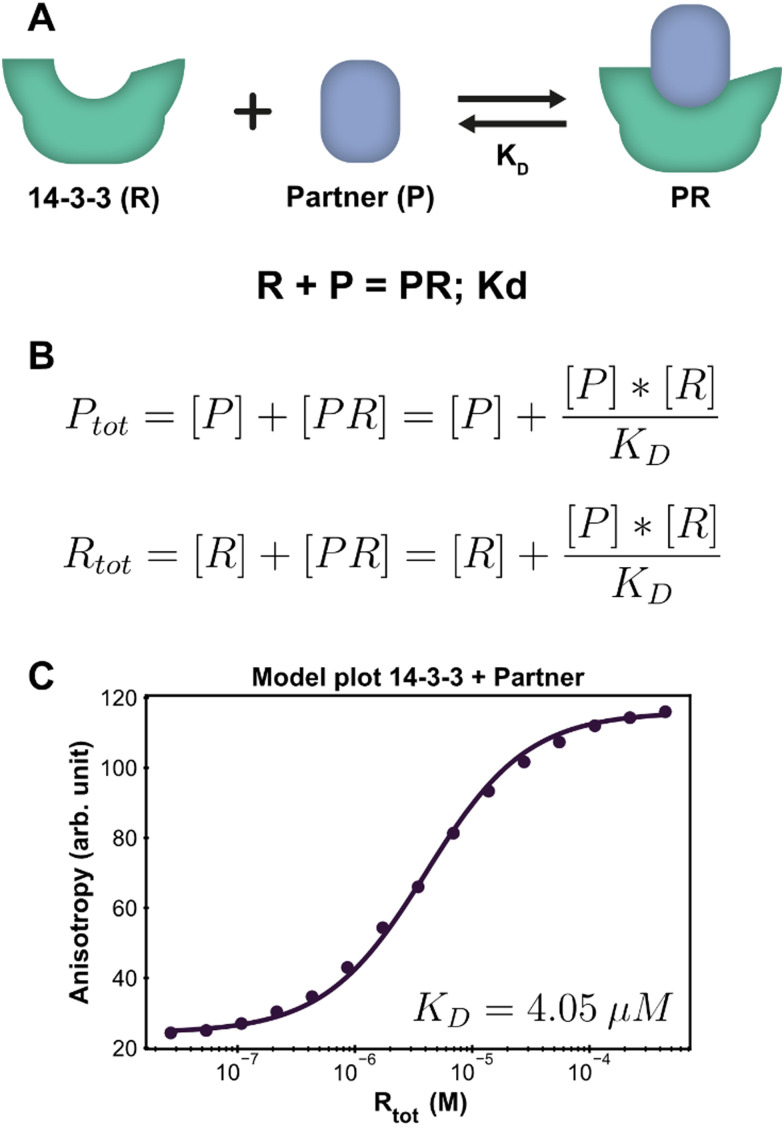
Application of the framework on the PPI system. (A) System description, each 14-3-3 protein (R) can complex with one partner (P) protein (TASK3). The model builder input corresponding to this reversible reaction is displayed beneath the cartoon. (B) The equilibrium equations determined for this system. (C) Fluorescence anisotropy data collected for this system^26^ (dots are the averages of the experimental technical repeats) in the presence of 10 nM P (labeled component) together with the model fit (line).

The PPI example system consists of a single reversible reaction and the corresponding system description is depicted in Fig. 2A. To create a custom model for this PPI, the reaction was entered in the form ‘complexing species = complex; dissociation constant’. From this, the equilibrium equations in Fig. 2B are automatically generated and simplified, such that the final equations only consist of component concentrations and equilibrium constants. The ESI† contains an overview with the exact input values for each step to model the system discussed here, which can be used as a tutorial reference (ESI† Appendix, Section 3).

Parameter fitting and additional analysis is performed from the framework main script, which offers a simplified interface to the underlying functions. It is also possible to directly execute the functions in the framework for advanced customization if desired. For this simple PPI system, only the model plot analysis is executed. The framework determines a point estimate for the fit parameter *K*_D_ before performing any chosen analyses. It is important to consider that the optimal estimate is determined within the constraints of the custom model and the framework thus does not judge the correctness of the system description, *i.e.*, the specified equilibrium reaction(s). In order to validate the proposed system description, we inspect both the obtained parameter value and the model plot together with the root mean square error (RMSE) of the model fit through the data. The calculated estimate for the *K*_D_ is 4.05 μM, which is in line with previous findings.^37^ The chosen analysis visualizes all the experimental data and the model prediction for each titrate (14-3-3) concentration using the determined point estimate (Fig. 2C). Inspecting the graph shows that the model prediction accurately describes the data with the determined parameter value, something that is corroborated by the RMSE, with a value of 1.56, being small compared to the fitted experimental data values.

### Molecular glue for PPI stabilization

**Fig. 3:**
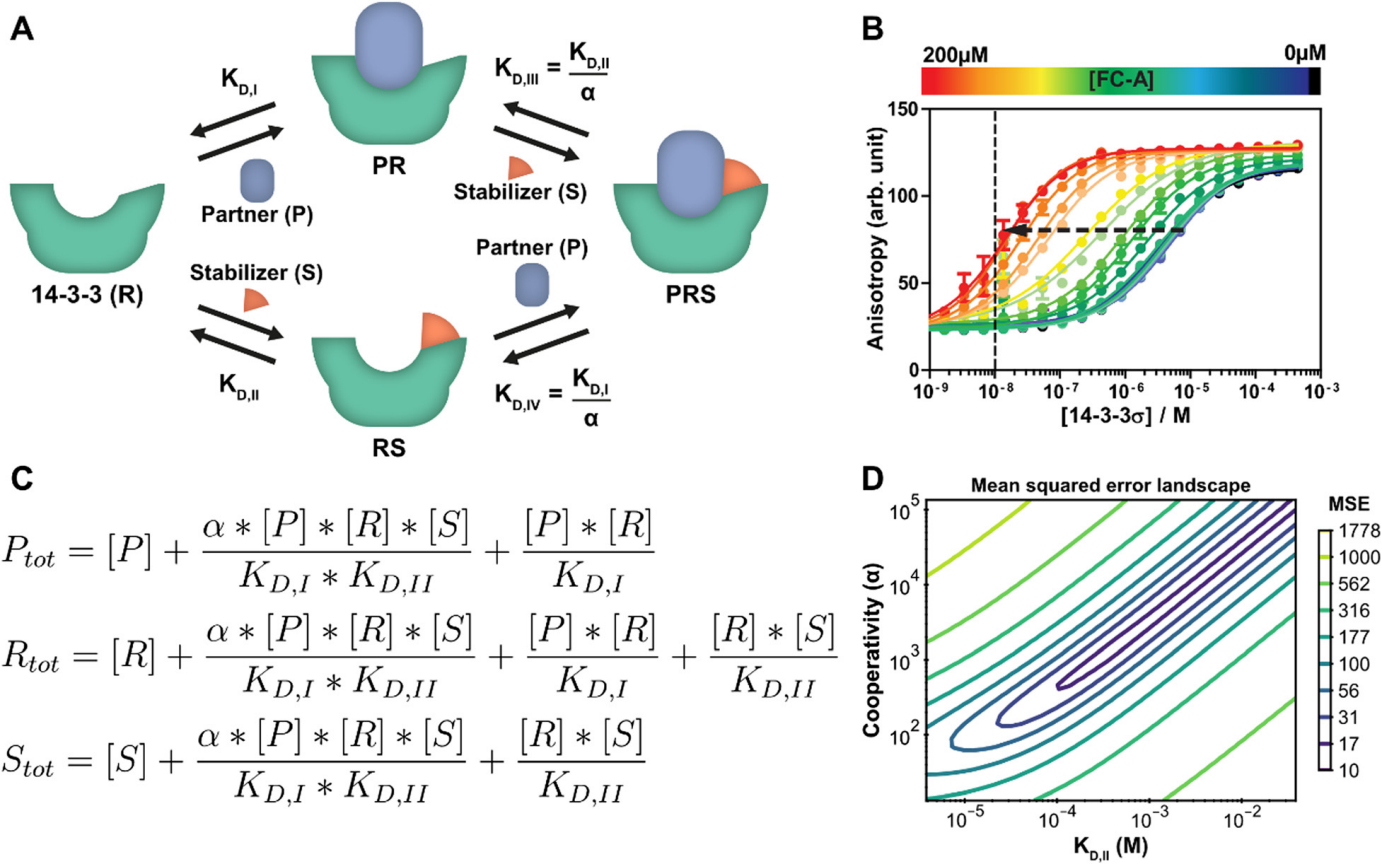
Protein–protein interaction stabilization case. (A) System description: the adapter protein 14-3-3 (R) binds to its partner TASK3 (P) with dissociation constant *K*_D,I_. In the presence of fusicoccin-A (S), the interaction is stabilized by a factor *α*, reducing the apparent affinity. Either the partner or stabilizer can bind first to form the ternary complex. (B) Fluorescent polarization data in the presence of varying concentrations of fusicoccin-A and a partner (labelled component) concentration of 10 nM (line), previously published^26^ for the model created specifically for this system. Note the decrease in EC_50_ value with the addition of the stabilizer (arrow). (C) Equilibrium equations automatically determined by the framework for this system. (D) Error-landscape plot centred on the determined estimates. The contours show that there is a valley of parameter combinations that result in a relatively low mean squared error (MSE).

Stabilization of PPIs, for example with PROTACs or molecular glues, is a highly topical research field in chemical biology and drug development.^3,4^ Molecular glues have proven to be an emerging and versatile strategy for 14-3-3 proteins as well.^40^ At its core, the PPI between two proteins is selectively stabilized by way of a third, low molecular weight compound.^33^ Besides the affinity of the drug compound for the protein targets, the cooperativity of the ternary complex formation is also a critical optimization parameter. A schematic representation of such a ternary complex formation with a molecular glue, which is an extension of the protein–protein interaction of the previous section, can be seen in Fig. 3A. While it is still possible for 14-3-3 (R) to directly bind to the partner protein (P), an additional path is now available where a stabilizing molecular glue binds first. As a result, the affinity for the partner is greatly increased. Because of the thermodynamic cycle in this system, it can be described entirely using the individual binding affinities (*K*_D,I_, *K*_D,II_) and one cooperativity factor (*α*).^26^ Though models have been derived for such similar ternary systems before,^17,20^ we here show how our framework can be used to automatically derive and analyze such a model. In this specific system, the stabilizer molecular glue (S) does not interact significantly with the partner protein by itself, in contrast to for example PROTACs, but such an additional reversible reaction could be added straightforwardly.

A custom model is created for the system description displayed in Fig. 3A from which the framework derives the equilibrium mass balance equations. For this three component system, this results in the set of three equations shown in Fig. 3C with the equilibrium concentrations of the three free components as the three unknowns. The experimental data for this case consists of 2D fluorescence anisotropy titrations (Fig. 3B and Dataset S2, ESI†).^26^ The step-by-step guide for this system can be found in ESI† Appendix, Section 4. Since the *K*_D,I_, (4.05 μM) was already determined in the previous section, this parameter is entered as such and kept constant and the model solver determines point estimates for the *α* and *K*_D,II_ parameters, resulting in values of 1.34 × 10^3^ and 0.389 mM respectively. These values are in good agreement with the previously determined values of 1 × 10^3^ and 0.3 mM.^26^

To provide additional context and insights on the cooperativity value *α* and the affinity of the compound for 14-3-3 (*K*_D,II_), the mean squared error landscape around the determined point estimates was visualized (Fig. 3D). The landscape gives a sense of the interdependence of these two parameters and the influence of each by displaying contour lines. A valley of parameter combinations results in a relatively low mean squared error. The *K*_D,II_ and *α* values display positive correlation, increasing both values (weaker initial binding, stronger cooperativity) still results in a relatively good prediction of the input data. Nevertheless, starting the solver from several combinations found at the valley of the landscape as initial guess values results in the same point estimates, indicating that there is a preference for the determined estimates over the other possible combinations. A sharp rise in error can be observed when one of the parameters is fixed and the other is varied. This indicates that the ratio of these parameters is important for accurate model prediction and can thus be determined with high confidence. Next to that, this analysis indicates that optimization regarding either *K*_D,II_ or *α* are both valid approaches for improvement of compound properties.^41,42^

While a point estimate is the best single-value approximation of a parameter, a confidence interval can be effective to get a sense for the certainty (or spread) of the reported estimate. The framework can determine this interval using a nonparametric, bias corrected bootstrap approach.^43–45^ As an example, the confidence interval analysis is performed with 2000 repeats in order to get an appropriate sample size and a 95% confidence interval (ESI,† Fig. S6). Additional information on the bootstrap method is available in ESI† Appendix, Section 5. The confidence interval (lower; median; upper) for the *K*_D,II_ (0.189 mM; 0.392 mM; 2.19 mM) and the *α* (0.687 × 10^3^; 1.34 × 10^3^; 7.35 × 10^3^) show an order of magnitude difference between the limits of the confidence interval. This broad range corresponds with the stretched valley in Fig. 3D and is important to consider when drawing conclusions from parameter estimates.

### Multicomponent immunodiagnostics platform

**Fig. 4:**
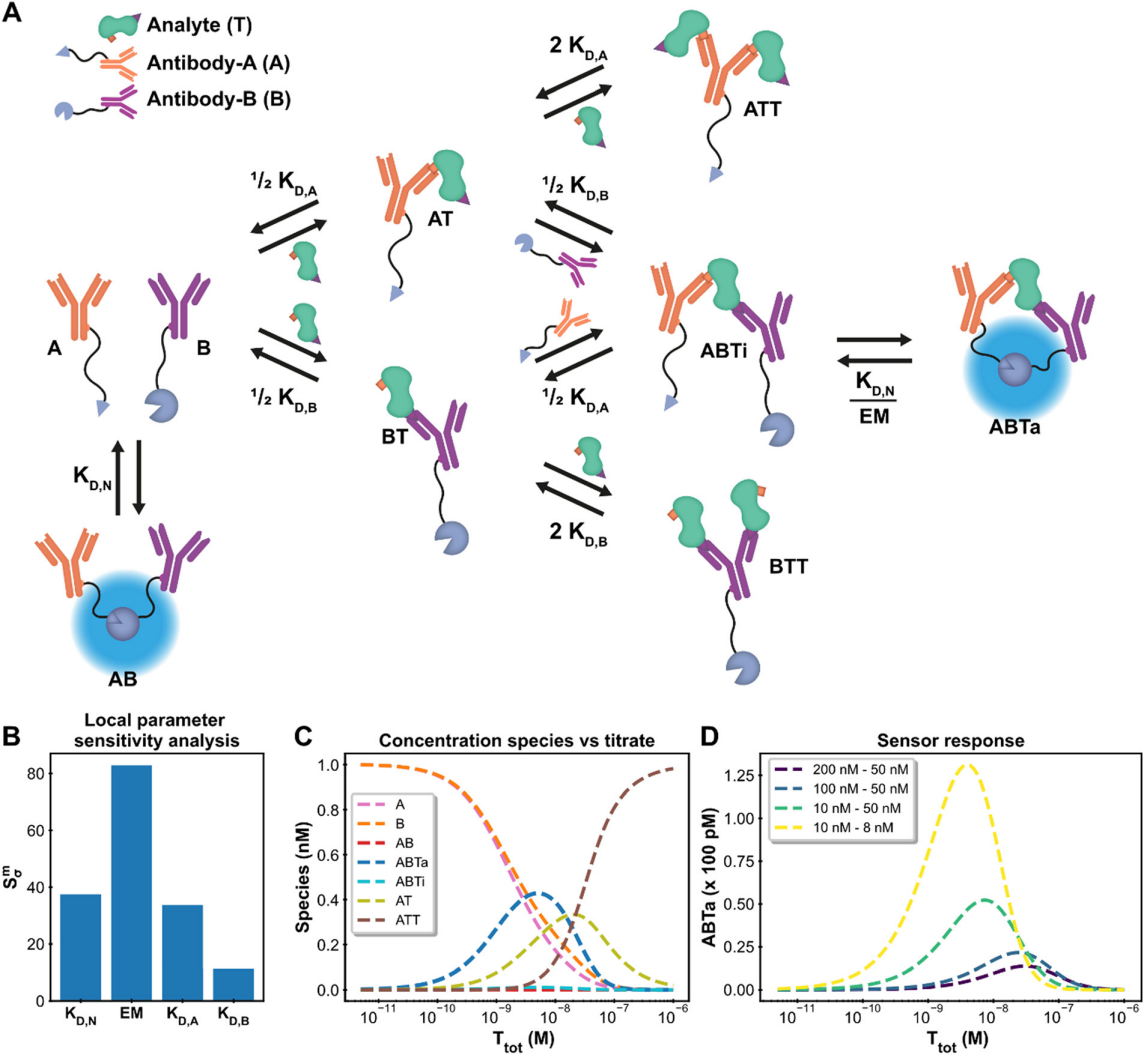
Ratiometric Plug-and-Play Immunodiagnostics (RAPPID) case. (A) System description.^25^ The antibodies A and B are both conjugated to a part of split NanoLuc luciferase, which emit light upon complex formation. The antibodies are specific for a certain analyte but target different epitopes. Upon antibody-analyte sandwich formation, the effective molarity for the split luciferases increases, resulting in increased complex formation and consequently increased signal. (B) Local parameter sensitivity analysis for the system using cardiac troponin I data (and corresponding concentrations) and a perturbation of +50%. The reference values are the point estimates determined by the framework. (C) Concentration plot generated with framework displaying how each concentration changes as the total titrate concentration increases. This analysis is performed without any experimental data with the fixed parameter values *K*_D,N_: 2.5 μM, *K*_D,A_: 10 nM *K*_D,B_: 15 nM, EM: 100 μM, [*A*]_tot_: 1 nM, [*B*]_tot_: 1 nM. The species BT and BTT have been omitted for clarity. (D) Simulation of shift in active ternary complex (ABTa) concentration for different combinations of antibody affinities and an EM value of 10 μM. Both antibodies have a total concentration of 1 nM.

Antibodies (AB) are hugely important in contemporary chemical biology, drug discovery, molecular diagnostics and alike. Their bivalent nature complicates simple model description of AB binding events, especially in case where multiple ABs and analytes are involved, such as in immunodiagnostics. The Hook-effect is another highly important parameter to take into account, not only for assay design, but also for effective AB therapies.^46^ The recently developed Ratiometric Plug-and-Play Immunodiagnostics (RAPPID) platform facilitates the development of ratiometric bioluminescent immunoassays for a wide range of biomolecular targets.^25^ RAPPID combines the use of antibodies with split NanoLuc luciferases^47,48^ to detect the formation of sandwich immunocomplexes in solution. A schematic overview of the general system is depicted in Fig. 4A. The system consists of two types of antibodies (A and B) and an analyte (T). Each antibody binds to a different epitope (with affinities *K*_D,A_ and *K*_D,B_) which allows for the formation of the ABTi(nactive) sandwich. The two parts of the spilt NanoLuc have a designed affinity of *K*_D,N_. The increased effective molarity (EM) within the ternary complex promotes the subsequent complementation into the ABTa(ctive) NanoLuc complex, which then emits light. Statistical factors are also present in this model to derive the dissociation constants *K*_D,A_ and *K*_D,B_ as there are two possible binding sites between each bivalent antibody and the monomeric target.^25,32^ At higher analyte:antibody ratios, the formation of analyte–antibody–analyte complexes becomes predominant over the formation of the functional ternary complex, giving rise to the hook effect. This highly complicated chemical biology system, encompassing 10 single or multimolecular species, harbours many of the intricacies encountered in AB research and thus acts as an ideal context to show the framework's capacity for extended systems and advanced analyses. In addition, the RAPPID example demonstrates how the custom model can guide system engineering and influence experimental design.

The system description for the multicomponent immunodiagnostic platform for intensiometric detection of cardiac troponin I is displayed in Fig. 4A and a step-by-step guide (ESI† Appendix, Section 6) is also available. A total of eight reversible reactions describe this system. The measured data (ESI† Appendix, Dataset S3 and Fig. S8) is proportional to the concentration of formed active ternary complex (ABTa) multiplied by an unknown scaling factor, representing the enzymatic activity of the luciferase. This data-concentration relationship is not available as a default data function in the model builder. However, the framework is not limited to these predefined functions, and it is possible to easily specify the relationship. In the model builder we define the custom function: ABTa × *Scaling*. Instructions on custom data functions are available in ESI† Appendix, Section 7. With the model point estimates for the parameters *K*_D,A_, *K*_D,B_ and *Scaling* could be determined, with fixed values for *K*_D,N_ and EM. The values for *K*_D,A_, *K*_D,B_ and Scaling of 533 nM, 15.3 nM and 5.53 × 10^17^ RLU/M are identical to the values determined in the original paper.^25^ The framework thus also allows modelling of complicated multicomponent systems and with equal accuracy as specifically designed models.

In such complex systems, usually not all parameters contribute equally to the final measurement outcome. The influence of each parameter can be determined using local parameter sensitivity analysis.^49^ This quantifies the change in a given function (designated M) based on a percentage change in parameter value. The default M-function measures the change in the sum of squared errors between the model prediction and the experimental data. Parameters with high sensitivity greatly affect the final model prediction and their identification allows for focused properties optimization. Fig. 4B displays the sensitivity for the parameters in the cardiac troponin I model after a 50% increase in the parameter value. For this Immunodiagnostics system, the sensitivity of *K*_D,A_ is larger than that of *K*_D,B_. The weaker binding affinity of the A-antibody, as compared to that of the B-antibody, is the limiting parameter in the formation of the active complex. In addition, both the *K*_D,N_ and the EM parameters, which were fixed parameters in the original analysis, have greater sensitivity than the fitted parameters (*K*_D,A_, *K*_D,B_). A potential smaller error in the fixed values of sensitive parameters can thus strongly affect the determined estimates for the other parameters. The analysis thus not only identifies critical parameters, but also shows that obtaining accurate values for sensitive parameters is crucial and prompts experimental setups to be adjusted to suit these criteria. Another available analysis which measures the sensitivity for the maximum amount of active complex formation is given in ESI† Appendix, Section 8.

For the design of new immunodiagnostics, or molecular sensors in general, it is important to engineer the detection regime over a large range of possible analyte concentrations. Within the RAPPID system, the specific antibodies and the concentrations of the sensor components can be relatively easily adjusted in order to tune the sensor. While it is possible to gather new experimental data to explore for suitable sensor concentration regimes for specific analyte concentrations, it is more (cost-) efficient to use the framework to make predictions first. We use the concentrations analysis for this purpose. In this analysis, the concentrations of all species in the system over a range of titrate values of the analyte is visualized (Fig. 4C). The final assay output for a number of different antibody affinity combinations can be seen in Fig. 4D. The analysis reveals that the peak of the graph, where the greatest increase in luminescence signal is observed, is dictated mostly by the antibody affinity. Greater affinities also increase the total signal as can be observed from the figure. The maximum complex formation can be tuned by changing the total antibody concentration. The analysis allows the selection of antibodies that are most suited for the intended analyte concentrations at a fraction of the time or costs necessary to perform the experimental measurements.

While the system description of the immunodiagnostics platform in Fig. 4A is already highly complex, it actually still omits several other higher-order species that could potentially be formed. When building a custom model by hand for a more elaborate multicomponent system it is not uncommon to exclude complexes that are assumed to form in negligible amounts. This is in part because manually deriving the equilibrium equations for these extremely large systems becomes very labour intensive and error prone. Using our framework approach it is possible to easily extend any multicomponent system within minutes with additional equilibria and new species. This allows for proper and rapid, validation of the role and relevance of such higher-order complexes. An extended system description for the RAPPID system was therefore modelled in ESI† Appendix, Section 9. This extended custom model contains 8 additional equilibria, additional to the 8 equilibria already shown in Fig. 4A. Analysis of the modelling of the data with this extended custom model revealed that several additional higher-order four-component complexes (the two antibodies A and B bound to two analyte molecules T simultaneously) can be formed in significant amounts at specific, higher analyte concentrations. The analysis thus brings forward that, depending on analyte concentrations, such complexes should potentially not be ignored. Fitting parameters to the extended custom model results in the point estimates *K*_D,A_ = 46.7 nM, *K*_D,B_ = 9.71 nM and scaling = 3.75 × 10^16^ RLU/M. These values have up to an order of magnitude difference compared to the previous estimates with the more simple model. This shows that the framework is not only capable of modelling complex systems, but also facilitates simple and fast validation of existing models.

## Conclusions

Equilibrium models can provide a wealth of information about chemical biology systems, but also other molecular systems such as those of the medicinal and supramolecular chemistry type. However, the creation of equilibrium models, including the derivation of equilibrium equations, takes expertise and a non-trivial amount of time. The framework approach presented here is capable of readily generating equilibrium models using an automatic derivation process for arbitrary molecular systems, defined by reversible reactions. A side-by-side comparison with three priorly published models for systems relevant in the field of chemical biology and of increasing complexity demonstrates the scope and accuracy of the framework. The parameter point-estimates determined by the framework closely matched or were identical to the values determined by the original models created solely for each of the systems in question. The framework can also be applied to gain insight into the quality of the determined estimates and the interrelationship of parameters in the model. Furthermore, building and analysing a computational equilibrium model using the framework can optimize experimental design and reduce the need for extensive experiments, even in situations without any prior experimental data.

The framework features an easy-to-use design that does not require a programming or mathematics background. Advanced users will find that the general structure and use of the Python programming language allows for straightforward extension and customization towards specific usage scenarios. The framework thus facilitates the simple and fast creation of effective computational equilibrium models in order to unravel, understand and delineate a broad range of molecular systems in chemical biology, such as molecular glues and antibodies, and beyond.

## Conflicts of interest

LB is scientific co-founder of the 14-3-3 biotech company Ambagon Therapeutics. All other authors declare no conflicts.

## Supplementary Material

CB-004-D2CB00211F-s001
